# Late-season corn stalk nitrate measurements across the US Midwest from 2006 to 2018

**DOI:** 10.1038/s41597-023-02071-9

**Published:** 2023-04-07

**Authors:** Anabelle Laurent, Alex Cleveringa, Suzanne Fey, Peter Kyveryga, Nathan Wiese, Mark Lefebvre, Darren Newville, Daniel Quinn, John McGuire, Haiying Tao, Thomas F. Morris, Fernando E. Miguez

**Affiliations:** 1 Department of Agronomy, Iowa State University, Ames, IA USA; 2 Research Center for Farming Innovation, Iowa Soybean Association, Ankeny, IA USA; 3 East Otter Tail Soil and Water Conservation District, Perham, MN USA; 4 Stearns County Soil and Water Conservation District, Waite Park, MN USA; 5 Department of Agronomy, Purdue University, West Lafayette, IN USA; 6 Simplified Technology Services LLC, Edgerton, OH USA; 7 Department of Plant Science and Landscape Architecture, University of Connecticut, Storrs, CT USA

**Keywords:** Plant sciences, Agriculture

## Abstract

The late-season Corn Stalk Nitrate Test (CSNT) is a well-known tool to help evaluate the after-the-fact performance of nitrogen management. The CSNT has the unique ability to distinguish between optimal and excessive corn nitrogen status, which makes it helpful for identifying the over-application of N so that farmers can adjust their future nitrogen decisions. This paper presents a multi-year and multi-location dataset of late-season corn stalk nitrate test measurements across the US Midwest from 2006 to 2018. The dataset consists of 32,025 corn stalk nitrate measurements from 10,675 corn fields. The nitrogen form, total N rate applied, US state, year of harvest, and climatic conditions are included for each corn field. When available, previous crop, manure source, tillage, and timing of N application are also informed. We provide a detailed description of the dataset to make it usable by the scientific community. Data are published through an R package and also available at the USDA National Agricultural Library Ag Data Commons repository and through an interactive website.

## Background & Summary

After seed costs, nitrogen (N) is the largest input cost in rainfed corn (*Zea maize* L.) production and is important for maintaining productivity and profitability. As N fertilizer can negatively impact the environment through losses into rivers, lakes, and the atmosphere, it is crucial to avoid excessive applications.

Most of the current or modern N fertilizer recommendations aim to estimate the gap between the N provided by the soil and N required by the plant^1^. However, N management is challenging as it is impacted by the uncertainty in predicting the various components of the N budget^2,3^. In addition to management practices and growing season information, to evaluate the nitrogen management, a well-known diagnostic tool, called late-season Corn Stalk Nitrate Test (CSNT), can help understand the after-the-fact performance of N management.

The CSNT measures the nitrate-N (NO_3_^−^) concentration in the 20 to 36 cm (8–14 inch) above-ground portion of corn stalks, which can be collected any time from about the 25% milk line stage of growth to 3 weeks after the black layer formation^4–7^. Nitrate taken up by a plant but not required to produce grain accumulates in this lower above-ground portion of the stalk. If the plant is unable to take up N due to drought conditions in the current growing season, the CSNT results can indicate that nitrogen uptake was excessive relative to plant nutrient needs (N supply vs. corn N demand). Thus, ideally the CSNT should be repeated over time due to the uncertainty associated with only one growing season.

The concentration values of nitrate in the lower corn stalk are usually categorized into four levels of N sufficiency for corn growth. Those categories were defined regarding the relationship between corn stalk nitrate and relative yield to the predicted maximum yield^4,8^. They are described as follows: (i) deficient (<250 mg NO_3_^−^-N kg^−1^ dry stalk), which indicates N was likely yield-limiting, (ii) marginal (250–700 mg NO_3_^−^N kg^−1^ dry stalk), which indicates that N availability was very close to the minimal amount needed to maximize grain yield, (iii) optimal (700–2000 mg NO_3_^−^-N kg^−1^ dry stalk), which indicates a high probability that the N availability was sufficient to maximize yield grain, and (iv) excessive (>2000 mg NO_3_^−^-N kg^−1^ dry stalk), which reveals a high probability that N supply was higher than necessary to maximize yield^4,8^. The CSNT has the unique ability to distinguish between optimal and excessive corn N status^9^, which makes it helpful for identifying over-application of N so that farmers can adjust their future nitrogen decisions^1^.

Here, we introduce a dataset based on field-guided CSNT surveys across the US Midwest. Data were collected from 2006 to 2018 and were part of different projects funded by USDA-Natural Resources Conservation Innovation Grants, by the Iowa Legislature through the Integrated Farm and Livestock Management Program of the Iowa Department of Agriculture and Land Stewardship; by the Environmental Defense Fund; by the Walton Family Foundation, by local Iowa soil and water conservation districts, by Indiana Soybean Alliance, and Indiana Corn Marketing Council and executed by the Iowa Soybean Association On-Farm Network, the Environmental Defense Fund’s NutrientStar Field Testing Network (now called The Amplify Network, Ohio), Indiana State Department of Agriculture, INField Advantage Program, Purdue University Extension, local Indiana soil and water conservation districts, and local groups in Minnesota.

**Fig. 1:**
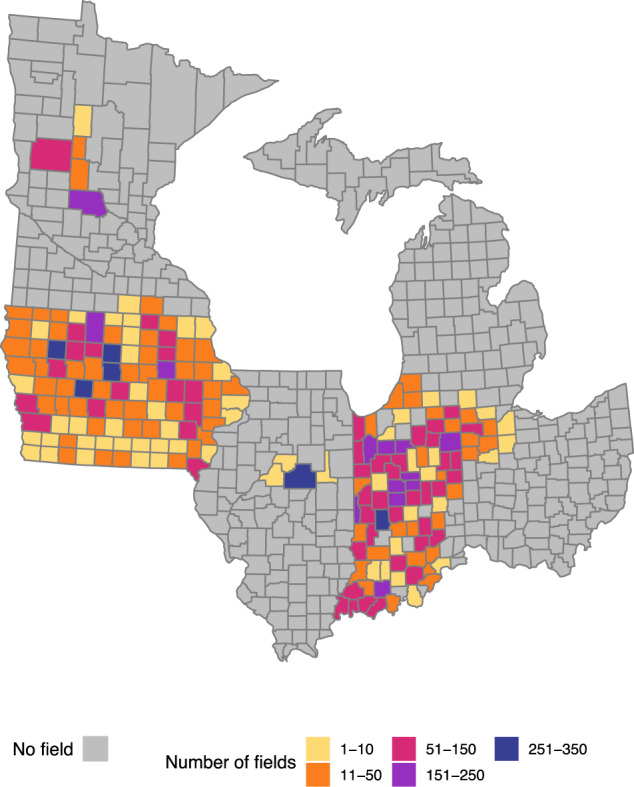
Number of field-guided surveys (i.e., fields) across the US Midwest at the county level where corn stalk nitrate data were measured.

In total, measurements from 10,675 corn fields were collected across six states in the Midwest (Fig. 1). The dataset contains 32,025 corn stalk nitrate measurements. Nitrogen form (commonly referred to as N source^10^) and the total N rate applied, US state, year of harvest, and climatic conditions are included for each site-year (trial location by growing season combination). When available, previous crop, manure source, tillage, and timing of N application are also included. Part of the dataset has been previously reported in peer-reviewed publications, but not made available^3,11–14^. The data have been published in the USDA National Agriculture Library Ag Data Commons repository at data.nal.usda.gov^15^. We also provide an R package, called *onfant.dataset*^16^, to store and easily update the dataset in the future by adding results of new field-guided surveys. In addition, we developed an online tool to interact with the dataset and provide descriptive statistical summaries.

## Methods

We obtained data from four different sources that were prepared as part of a joint USDA NIFA grant between the Iowa Soybean Association and Iowa State University. The first one is a dataset collected from 2007 to 2017 by the Iowa Soybean Association’s On-Farm Network from 4,211 fields located in all the counties of IA with a total of 12,633 CSNT measurements. The second dataset was created for a peer-reviewed paper^3^ and shared by the authors. These data were collected from 2008 to 2014 in Michigan, Ohio, Indiana, and Illinois. It contains a total of 3,208 CSNT measurements from 803 fields. In Illinois, most corn fields came from the same county (Fig. 1). The third dataset includes 21,219 CSNT measurements collected from 2011 to 2018 in Indiana in 5,306 fields. The fourth dataset includes 1,065 CSNT data collected from 2010 to 2012 in Minnesota in 355 fields located mostly in the central region (Fig. 1).

Using site coordinates, we retrieved weather data (i.e., temperature, rainfall, growing degree day, and solar radiation) from Iowa Environmental Mesonet Reanalysis using the apsimx R package^17,18^. The weather data are available in the R package *onfant*.dataset and have been published to the USDA National Agriculture Library Ag Data Commons (USDA NAL ADC)^15^ repository. As the coordinates were not available for the fields in Indiana, we used the township or county information. For privacy purposes, site latitude and longitude are not reported in the dataset. For map displays we added noise to the coordinates. We eliminated fields having at least one CSNT sample missing or when the total N rate applied was not reported.

**Fig. 2:**
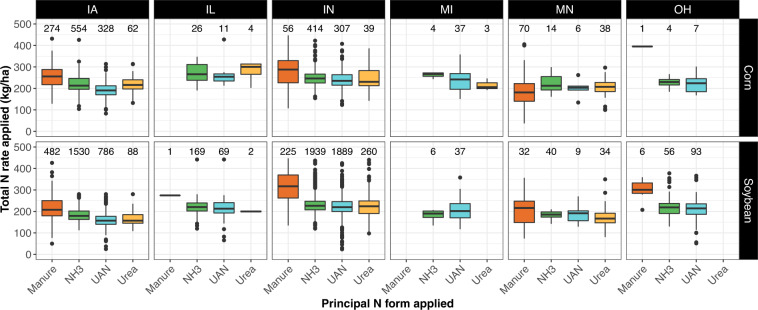
Distribution of the total N rate for the most representative N fertilizer form applied per previous crop and US state. Number of corn fields is displayed at the top of the corresponding boxplot. Only data for fields having a total N rate <450 kg/ ha are displayed (see Technical Validation).

The total N rate of different principal N forms applied shows contrasting distribution patterns across states and previous crops (Fig. 2). The median total N rate applied is lower for soybean as a previous crop than for corn as previous crop for most of the principal N forms applied and states. The median value of manure (here, all manure types combined) applications tends to be higher than the median values for commercial fertilizers, NH_3_, UAN, and urea, N forms for a specific state and previous crop (Fig. 2).

**Fig. 3:**
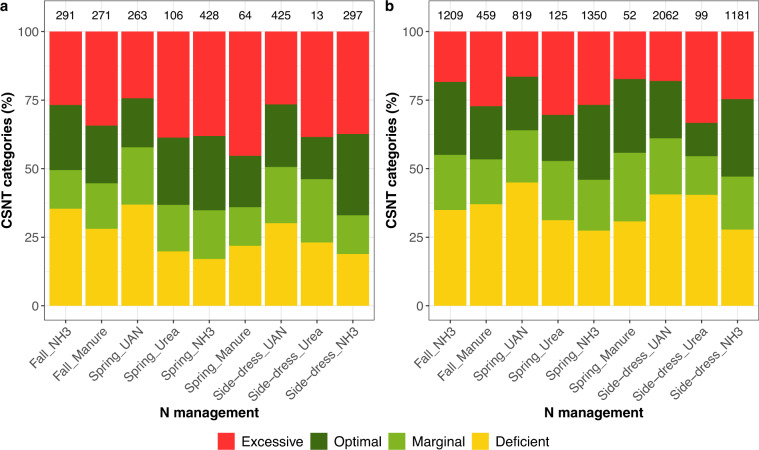
Percentage of CSNT categories per N management (a combination of application timing and N form) for corn following corn (**a**) and corn following soybean (**b**) rotations. N management with missing information about the application timing or N form are not represented. Number of corn fields per N management is displayed at the top of the corresponding bar. Only data for fields having a total N rate <450 kg/ ha are displayed (see Technical Validation).

Fields with corn as a previous crop had a higher percentage of excessive CSNT category and a lower percentage of deficient CSNT category than with soybean as a previous crop (Fig. 3).

**Fig. 4:**
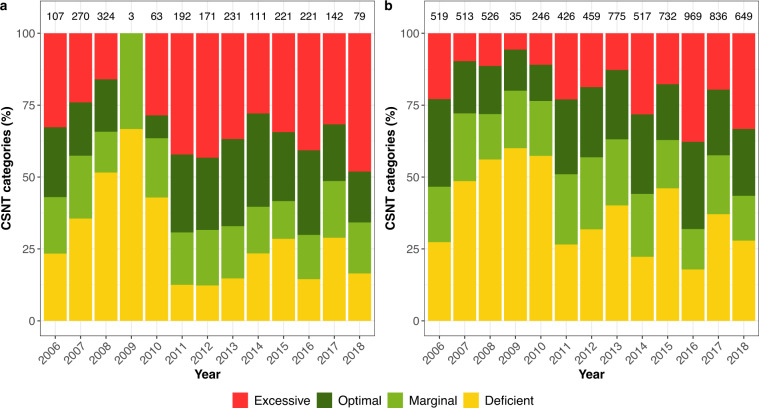
Percentage of CSNT categories per year for corn following corn (**a**) and corn following soybean (**b**) rotations. Number of corn fields per year are displayed at the top of the corresponding bar. Only data for fields having a total N rate <450 kg/ha are displayed (see Technical Validation).

The percentage of CSNT categories differed over time (Fig. 4). In 2008 and 2009 the percentage of the deficient category was over 50% for corn and soybean as previous crop.

## Data Records

Data have been published to the USDA National Agriculture Library Ag Data Commons (USDA NAL ADC)^15^ repository with an assigned Digital Object Identifier (dataset DOI: 10.15482/USDA.ADC/1527976). The complete dataset is also available as an R package *onfant.dataset*^16^ (ON-FArm Nitrogen Trials) that can be obtained from GitHub (https://github.com/AnabelleLaurent/onfant.dataset). Thus, the dataset can be subsequently exported as csv, xlsx or other similar tabular format. The R package contains four subsets of the whole dataset: one subset per dataset source.

The names, units, and descriptions of the columns are shown in Table 1.

- “Field_ID” records the unique identifier of a corn field with CSNT measurements.
- “Sample_number” corresponds to one CSNT measurement. Three samples were taken per “Field_ID” based on primary soil types in the field; thus, the number 1, 2 or 3 are expected.
- “Year” indicates the year of CSNT measurement.
- “State” reports the state where the corresponding “Field_ID” was located.
- “County” reports the county where the corresponding “Field_ID” was located.
- “County_centroid_latitude” and “County_centroid_longitude” indicate the county centroid latitude and longitude, respectively, where the samples were taken within a field. Due to privacy protection, the exact coordinates are not shared; consequently, we retrieved those two variables for the dataset publication.
- “Previous_crop” is the crop produced before the year of measurement. The main previous crops are soybean and corn. Some less frequent previous crop categories are hay, sorghum, wheat, and potatoes. If the original information reported “Other”, we kept the same label for the entry.
- “Manure_type” refers to the type of manure applied as organic nitrogen fertilizer such as poultry manure, swine manure or beef manure, for example. If no manure was applied, the “Manure_type” is set to “No manure”.
- “Manure_application” indicates whether each entry received manure (labeled as “Yes”) or not (labeled as “No”). Overall, 88% of the entries are labeled as “no”.
- “N_fertilizer_form” described the principal N form applied with inorganic or organic N fertilizers. These are urea, NH_3_ (anhydrous ammonia) or UAN (Urea Ammonium Nitrate). If the principal N form applied is manure, then the entries correspond the “Manure_type”. If the original information reported “Other”, we kept the same label for the entry.
- “N_fertilizer_form_simplified” is created from “N_fertilizer_form” where all the manure types are labeled as “Manure”. We kept labels “UAN”, “NH_3_”, and “Urea”, and all other N form types are labeled as “Other”.
- “N_application_timing” refers to the N timing application (e.g., Spring, Fall, Side-dress (early season). Some labels such as “Summer” or “Winter” occur a small number of times.
- “N_application _timing_simplified” is created from “N_application_timing” where we kept the labels “Fall”, “Spring” and “Side-dress”. Other entries are labeled “Other”.
- “N_management” is the combination of “N_fertilizer_form” and “N_application_timing”.
- “N_management_simplified” is the concatenation of “N_fertilizer_form_simplified” and “N_application_timing_simplified”.
- “Tillage_use” indicated whether each entry had tillage (labeled as “Yes”) or not (labeled as “No”)
- “Total_N_rate_lbac” refers to the total N rate applied during a corn growing season from all forms and timing, including the principal “N_fertilizer_form”. The total N rate unit is the pound per acre (lb/ac) as informed originally.
- “Total_N_rate_kgha” is a conversion of the “Total_N_rate_lbac” to kilogram per hectare. For corn, 1 lb/ac equals 1.12 kg/ha^19^.
- “Stalk_nitrate_N” is the main data record in the dataset. It corresponds to nitrate-N concentration in ppm using the CSNT.
- “GM_ppm” refers to the geometric means of stalk nitrate-N calculated from the three samples per field.
- “GM_4_category” expresses the corn N status into four N sufficiency categories (marginal, deficient, optimal, excessive) as described in the background section.
- “GM_2_category” expresses the corn N status into two N sufficiency categories: sufficient (including the “GM_4_category” marginal, optimal, excessive) and deficient otherwise.

**Table 1:** Description of the variables.

Variable	Description	Missing Values	Levels/Range	Additional Information	Example
Field_ID	Field ID	0	Levels: 10,675	Unique for each field	GSS2010OHMM015
Sample_Number	Sample number		Range: 1–3		1
Year	Year	0	Range: 2006–2018		2006
State	State	0	Levels: 6	IA, IL, IN, MI, MN, OH	IA
County	County name	15	Levels: 172		Allen
County_centroid_latitude	County of the field centroid latitude	144	Range: 38.0–47.1		41.64752
County_centroid_longitude	County of the field centroid longitude	144	Range: −96.2 - −83.6		−94.73380
Previous_crop	Previous crop	366	Levels: 18		Soybean
Manure_type	Manure animal source	0	Levels: 7	No manure and 7 animals	Swine Manure
Manure_application	Was manure applied	0	Levels: 2	Yes, No	Yes
N_fertilizer_form	Nitrogen fertilizer form	0	Levels: 16		Urea
N_fertilizer_form_simplified	Simplified N Form	0	Levels: 5		UAN
N_application_timing	Nitrogen fertilizer timing	1905	Levels: 8		Spring
N_application_timing_simplified	Nitrogen fertilizer timing	1905	Levels: 5		Fall
N_management	Combined Timing and N_form	0	Levels: 44		Fall_Swine Manure
N_management_simplified	Combined Timing_simplified and N_form_simplified	0	Levels: 17		Fall_Manure
Tillage_use	Whether tillage was done or not	726	Levels: 2		no-till
Total_N_rate_lbac	Total N rate	0	Range: 4–780 (lb/ac)		117
Total_N_rate_kgha	Total N rate	0	Range: 4.5–873.6 (kg/ha)		131.04
Stalk_nitrate_CSNT	Stalk Nitrate-N	0	Range: 1–36,700 (ppm)		343
GM_ppm	Stalk nitrate geometric mean of each field	0	Range: 1–16,326 (ppm)		348.8
GM_4_category	4 categories of GM	0	Levels: 4	Deficient, Marginal, Optimal, Excessive	Marginal
GM_2_category	2 categories of GM	0	Levels: 2	Deficient, Sufficient	Sufficient

We developed and launched an interactive web tool called ONFANT (https://onfant.agron.iastate.edu/) using R Shiny^20^. ONFANT is accessible for any user without restrictions on permission such as IP address. ONFANT uses a friendly interface enabling users to explore the dataset having access to descriptive displays and statistical summaries. Users can interact with the dataset by selecting variables and filtering specific factor levels. For example, users can explore data related to a specific state, filter by previous crop, or chose the total nitrogen unit (kg/ha or lb/ac). We displayed an interactive map of corn field locations colored by N form type, manure application, N application timing, CSNT category, and tillage. Due to privacy concerns, the exact coordinates are not identifiable at the current zoom level on the map.

We also encourage users to use the help functions available in the R package *onfant.dataset* to have access to specific variable’s description. In the future, the R package *onfant.dataset* and the interactive web tool ONFANT (https://onfant.agron.iastate.edu/) might be updated with additional data, new visualization features, and statistical summaries.

## Technical Validation

Each dataset was carefully examined several times by two persons, and we paid special attention to the value of the total N rate provided by a farmer or crop consultant. We performed a systematic examination of the variable’s labels and value counts for the categorical variables to avoid spelling errors. We also ran basic statistics (e.g., mean, and interquartile values) to correct the mistyping information or checked for outliers by validating them in the original datasets. If possible, data providers were contacted if details within their raw data were unclear. For “Total_N_rate”, we plotted the frequency distribution and returned to the original articles for checking extreme values. We carefully inspected the consistency between the latitude and longitude variables and county/state names.

While the geometric mean of the CSNT was provided for some datasets, we computed this variable again using the raw data (nitrate-N in ppm) from the three samples to homogenize the computation method. The fields were discarded if they had at least one CSNT sample missing.

The dataset created for the peer-reviewed paper already included aggregated data, but we returned to the original files in case of unclear information or inconsistent observations.

Despite having access to weather for most fields, we retrieved monthly rainfall information again to have a uniform source of information for the corresponding variables.

If the total N rate applied was missing, the field was removed and not included in the final dataset.

Out of 10,675 corn fields, 80 had a total N rate higher than 450 kg/ha (400 lb/ac). As most of them had manure as the main N form applied, we suspect that the value informed is too high because of the use of inappropriate manure book values (total N content in % per manure dry matter) rather than manure nutrient analysis. For that reason, we only display data for fields with a total N rate lower than 450 kg/ha for Figs. 2–4.

## Usage Notes

One of the caveats of our dataset is that crop management information is not consistently informed for all fields. Field management variables have been simplified to be able to join the datasets together. Also, as yield data were not reported, we were not able to explore the relationship between yield and CSNT category or between yield and nitrogen application rate. Finally, as the exact field coordinates are not shared, due to privacy protection, the use of our dataset for crop growth models such as APSIM^21^ or DSSAT^22^ are limited.

## Data Availability

The dataset is easy to access by Microsoft Excel or other software like R^23^ or Python^24^. The complete dataset can be obtained from GitHub (https://github.com/AnabelleLaurent/onfant.dataset).
